# To drain or not to drain in colorectal anastomosis: a meta-analysis

**DOI:** 10.1007/s00384-016-2509-6

**Published:** 2016-01-30

**Authors:** Hong-Yu Zhang, Chun-Lin Zhao, Jing Xie, Yan-Wei Ye, Jun-Feng Sun, Zhao-Hui Ding, Hua-Nan Xu, Li Ding

**Affiliations:** Department of Gastrointestinal Surgery, The First Affiliated Hospital of Zhengzhou University, No. 1 East Jianshe Road, Erqi District, Zhengzhou, 450052 Henan Province China; Centre for Eye Research Australia, University of Melbourne, Royal Victorian Eye and Ear Hospital, Melbourne, Australia; Department of Cardiovascular Internal Medicine, The First Affiliated Hospital of Zhengzhou University, No. 1 East Jianshe Road, Erqi District, Zhengzhou, 450052 Henan Province China

**Keywords:** Drain, Colorectal anastomosis, Meta-analysis, Postoperative complications

## Abstract

**Background:**

Currently, many surgeons place a prophylactic drain in the abdominal or pelvic cavity after colorectal anastomosis as a conventional treatment. However, some trials have demonstrated that this procedure may not be beneficial to the patients.

**Objective:**

To determine whether prophylactic placement of a drain in colorectal anastomosis can reduce postoperative complications.

**Methods:**

We systematically searched all the electronic databases for randomized controlled trials (RCTs) that compared routine use of drainage to non-drainage regimes after colorectal anastomosis, using the terms “colorectal” or “colon/colonic” or “rectum/rectal” and “anastomo*” and “drain or drainage.” Reference lists of relevant articles, conference proceedings, and ongoing trial databases were also screened. Primary outcome measures were clinical and radiological anastomotic leakage. Secondary outcome measures included mortality, wound infection, re-operation, and respiratory complications. We assessed the eligible studies for risk of bias using the Cochrane Risk of Bias Tool. Two authors independently extracted data.

**Results:**

Eleven RCTs were included (1803 patients in total, 939 patients in the drain group and 864 patients in the no drain group). Meta-analysis showed that there was no statistically significant differences between the drain group and the no drain group in (1) overall anastomotic leakage (relative risk (RR) = 1.14, 95 % confidence interval (CI) 0.80–1.62, *P* = 0.47), (2) clinical anastomotic leakage (RR = 1.39, 95 % CI 0.80–2.39, *P* = 0.24), (3) radiologic anastomotic leakage (RR = 0.92, 95 % CI 0.56–1.51, *P* = 0.74), (4) mortality (RR = 0.94, 95 % CI 0.57–1.55, *P* = 0.81), (5) wound infection (RR = 1.19, 95 % CI 0.84–1.69, *P* = 0.34), (6) re-operation (RR = 1.18, 95 % CI 0.75–1.85, *P* = 0.47), and (7) respiratory complications (RR = 0.82, 95 % CI 0.55–1.23, *P* = 0.34).

**Conclusions:**

Routine use of prophylactic drainage in colorectal anastomosis does not benefit in decreasing postoperative complications.

**Electronic supplementary material:**

The online version of this article (doi:10.1007/s00384-016-2509-6) contains supplementary material, which is available to authorized users.

## Introduction

Currently, many surgeons routinely place a drain after the completion of colorectal anastomosis in case of postoperative complications, such as anastomotic leakage and wound infection. However, prophylactic drainage has remained controversial since it was firstly introduced by Theodore Billroth in 1877 [1]. The placement of a drain is expected to be an indicator of postoperative complications, which may help prevent hematoma, fluid collection, or abscess formation and minimize the severity of complication-related symptoms in colorectal surgery. Manz et al. reported that drains may warn doctors early about intraperitoneal hemorrhage or anastomotic leakage [2]. In addition, two studies have indicated that drainage of the presacral space could reduce the incidence of anastomotic dehiscence and pelvic sepsis [3, 4]. However, other studies demonstrated that drains may cause infection around the anastomotic area, affect anastomotic healing, and increase the incidence of anastomotic dehiscence [5–7]. Drains could stimulate the formation of fluid collection by causing a foreign-body reaction or inhibiting the closure of the dead space [8]. Berliner et al. found that drainage of the anastomosis increased leakage rate, morbidity, and mortality [9]. Surgeons are confused by the controversial conclusions, and most of them still use a drain in the abdominal or pelvic cavity after colorectal anastomotic surgery according to their personal experience.

To better provide practice guideline for surgeons, we performed this meta-analysis to determine whether the prophylactic placement of a drain after colorectal anastomosis could reduce postoperative complications.

## Methods

Our study was carried out according to the recommendations of the PRISMA statement [10].

### Literature search

A systematic literature search was performed up to June 24, 2015 using the terms “colorectal” or “colon/colonic” or “rectum/rectal” and “anastomo*” and “drain or drainage” in the following databases: MEDLINE, EMBASE, the Cochrane Library, the Controlled Clinical Trials Database, China National Knowledge Infrastructure (CNKI), and Wanfang Standards Database (WFSD). The reference lists of the identified relevant articles, conference proceedings, and ongoing trial databases were further screened for potentially relevant studies.

We excluded observational studies, quasi-randomized trials, crossover trials, and cluster-randomized trials. We did not exclude any study based on language of publication or publication status.

### Selection criteria

Titles and abstracts of all the identified articles were screened, and the trials were included according to the following criteria: (a) prospective randomized controlled trials (RCTs) that compared the routine use of prophylactic drainage of colonic and/or rectal anastomoses to no drainage; (b) patients with colonic or rectal tumor, diverticular disease, volvulus, and inflammatory disease located anywhere along the colon and the rectum that need to be treated with surgical re-section and anastomosis; and (c) outcomes included anastomotic leakage (clinical or radiologic or both; clinical leakage defined as discharge of feces or gas from a drain site or wound or localized or generalized peritonitis with tenderness, fever, and leucocytosis as well as surgical or radiological confirmation of a leak or confirmed by autopsy and radiologic leakage defined as which is detected in an asymptomatic patient by contrast medium enema), mortality (patients were followed at least 30 days), wound infection (defined as pus coming from the surgical wound), re-operation, and respiratory complications (defined as the production of purulent sputum with appropriate clinical and radiological changes). Studies were excluded if they were retrospective and had no control arm or data regarding the efficacy or complications.

### Data extraction and quality assessment

Two investigators (Zhang HY and Ye YW) independently extracted the following data from all the included trials: patient characteristics, study design, patient inclusion and exclusion criteria, preoperative preparation, procedure process, and incidence of postoperative complications. Details of randomization (generation and concealment), number of patients allocated to each group, type of drainage, site of anastomosis (intraperitoneal, extraperitoneal, or not specified), duration of drainage, and number of patients that lost follow-up were recorded. The methodological quality of each trial was assessed by the same reviewers. If there were any discrepancies, a third investigator (Xie J) was consulted and consensus was reached by discussion. The quality of each study was assessed using the Cochrane Collaboration’s tool for assessing the risk of bias, a value of “low risk,” “high risk,” or “unclear” was assigned to the seven domains: (a) random sequence generation, (b) allocation concealment, (c) blinding of participants and personnel, (d) blinding of outcome assessment, (e) incomplete outcome data, (f) selective reporting, and (g) other bias [11].

### Unit of analysis issues

We included only parallel group trials. If a study contained more than two groups, we fused two groups (by using the appropriate formula for adding the standard deviations when required) when we thought that they were equivalent according to the criteria of our protocol or separated them and split the control in half.

### Dealing with missing data

We contacted the authors for apparent missing data. We did not use imputed results. Data were entered as intention-to-treat (ITT) data as much as is feasible. If not, the study was quoted as at high risk of bias for selective reporting and then the data were entered on a per protocol basis.

### Statistical analysis

Statistical analyses were performed with the recommendations of the Cochrane Collaboration Guidelines [12]. The meta-analysis was performed using Review Manager Software (RevMan, version 5.3 for Windows) [11]. Dichotomous outcomes presented as relative risk (RR) and the 95 % confidence interval (CI) was quantified for all the analyses. Heterogeneity was assessed with Cochran’s χ^2^ test and the *Ι*^2^ test. Statistically significant heterogeneity was considered to be present when *P* was <0.10 and the *Ι*^2^ value was >50 % [13]. The fixed-effect model was used if there was no significant statistical heterogeneity (*P* > 0.10 and *Ι*^2^ < 50 %). If heterogeneity existed, the random-effect model was applied [14]. Funnel plots were drawn to help identify the publication bias.

Sensitivity analyses were performed with high quality of trials. Subgroup analyses were performed by stratifying the RCTs based on the site of anastomosis (intraperitoneal and extraperitoneal), the type of drainage (active drain with suction and passive drain without suction), and the race (Asian and European).

## Results

**Fig. 1:**
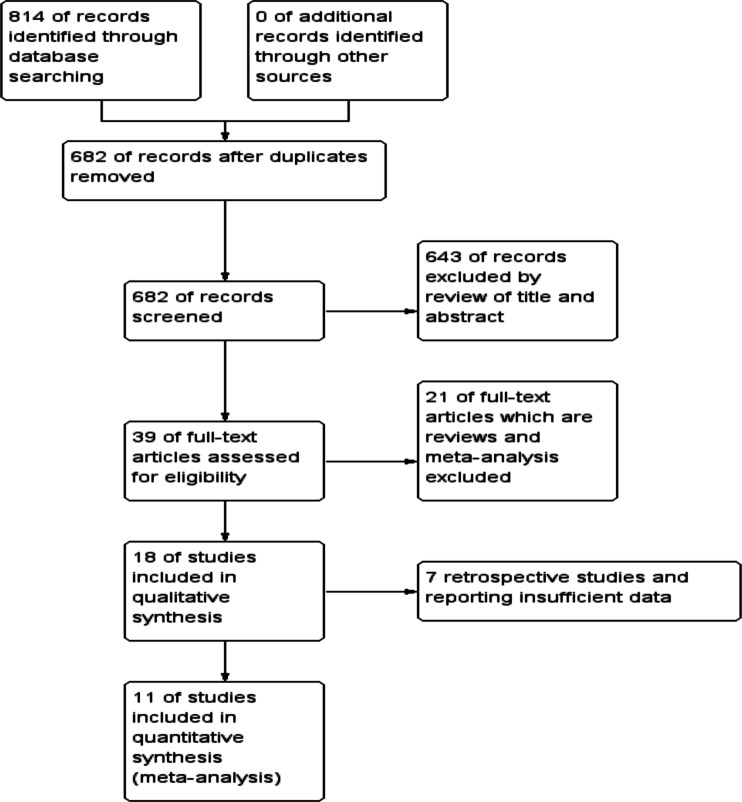
Flow diagram showing the selection of randomized controlled trials

In total, 814 potential relevant publications were identified; 18 trials were suitable for systematic review. We excluded seven retrospective trials with insufficient data. Eleven trials with 1803 patients met the inclusion criteria and underwent further analysis (Fig. 1).

The characteristics of the included RCTs are shown in Table 1. Of these 11 RCTs, seven were published in English [15, 18, 19, 21–24], two were published in German [17, 20], and two were published in Chinese [16, 25]. The study locations were in Singapore [15], Germany [17, 20], Denmark [18], the UK [19, 23, 24], France [21, 22], and China [16, 25]. The average age of patients in each trial was above 50 years (range 17–98 years). Enrolled patients were all diagnosed with tumor in three trials [15–17]. The other eight trials included patients with tumor, diverticular disease, inflammatory bowel disease, and others. The proportion of tumor patients was 78.1 % (1409/1803) among all patients. The type of drainage used was active in four trials (drain with suction) [15, 22–24], was passive in six trials (drain without suction) [16–20, 25], and both active and passive in one trial [21]. The length of drainage in all patients was less than 8 days. The sites of anastomosis of included trials were as follows (Table 1): four trials described intraperitoneal anastomoses [17, 18, 20, 21], two trials described extraperitoneal anastomoses [15, 24], and five trials included both [16, 19, 22, 23, 25].

**Table 1:** General characteristics of the included trials

Study	Number (D^+^/D^−^)	Age, years (D^+^/D^−^)	Male, % (D^+^/D^−^)	Multicenter/monocenter	Site of anastomosis	Type of anastomosis	Duration of drainage (day)	Type of drainage	Lost to follow-up
Brown [15]	31/28	66/64^a^	64.5/57.1	Mono	Extra	Stapled	3	Closed suction	1
Cao [16]	120/90	52.4/50.3^b^	70.8/65.6	Mono	Extra and intra	Stapled	5.6 ± 2.4^b^	Latex drain	0
Hagmuller [17]	60/53	NA	NA	Mono	Intra	NA	NA	Easy flow	0
Hoffmann [18]	28/32	72/73^b^	32.1/46.9	Mono	Intra	Stapled/hand-sewn	5	Corrugated latex drain	0
Johnson [19]	49/57	64.1/69.4^b^	40.8/50.9	Multi	Extra and intra	Stapled/hand-sewn	3 (1–8)^a^	Corrugated silastic drain	1
Mennigen [20]	51/48	NA	NA	Mono	Intra	NA	NA	Silicone	1
Merad [21]	156/161	67/67^b^	38.5/46.0	Multi	Intra	Stapled	≤5	Suction and non-suction	2
Merad [22]	248/246	66/66^b^	48.6/52.2	Multi	Extra and intra	Stapled/hand-sewn	≤5	Closed suction	2
Sagar [23]	94/51	66/70^a^	55.3/52.9	Mono	Extra and intra	Stapled/hand-sewn	3 or 7	Closed suction	3
Sagar [24]	52/48	58/64^a^	53.8/45.8	Multi	Extra	Stapled/hand-sewn	7	Closed suction	4
Zhang [25]	50/50	61/63^b^	66.0/58.0	Mono	Extra and intra	Stapled	7.3 ± 2.6^b^	Latex drain	2

^a^Median

^b^Mean

*NA* not available, *D* ^+^ drain, *D* ^−^ no drain, *extra* extraperitoneal, *intra* intraperitoneal

### Risk of bias in included trials

**Fig. 2:**
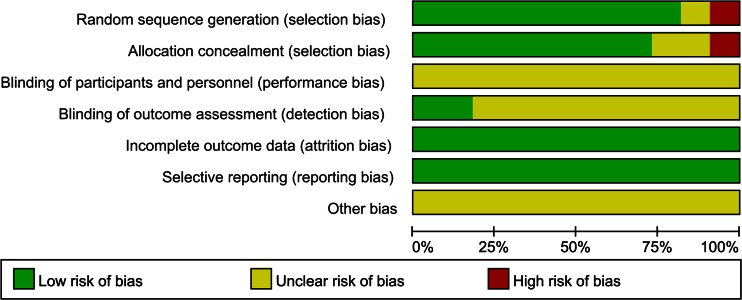
Risk bias of graph. Each risk of bias item presented as percentages across all of the included trials, which indicated the proportion of different level risk of bias for each item

**Fig. 3:**
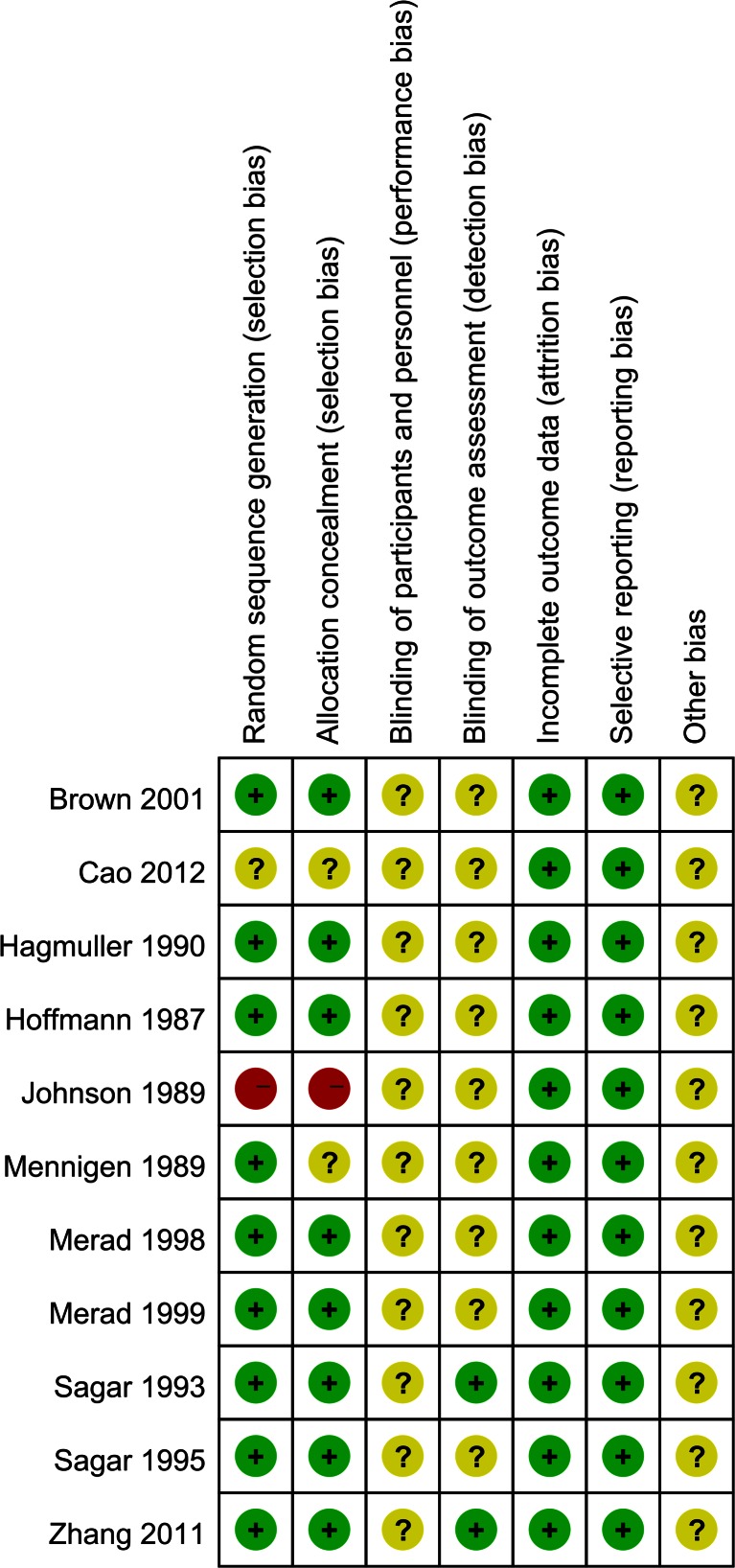
Risk bias of summary. Judgments about each risk of bias item for each included trials. *Green* indicates low risk of bias. *Yellow* indicates unclear risk of bias. *Red* indicates high risk of bias

The Cochrane Risk of Bias Tool was used to assess the potential risk of bias of the included trials in this meta-analysis (Figs. 2 and 3). Eight trials reported adequate random sequence generation and allocation concealment. Two trials stated adequate random sequence generation but unclear allocation concealment. However, one of them used the birth of patient to randomize [19]. One trial only mentioned randomization without detailed method [16]. Surgeon blinding would have been inappropriate in all of the included trials; two of the included trials blinded the outcome assessors [23, 25].

Of the 1803 patients enrolled in 11 trials, 939 were in the drain group and 864 were in the no drain group.

### Primary outcomes

#### Overall anastomotic leakage

**Fig. 4:**
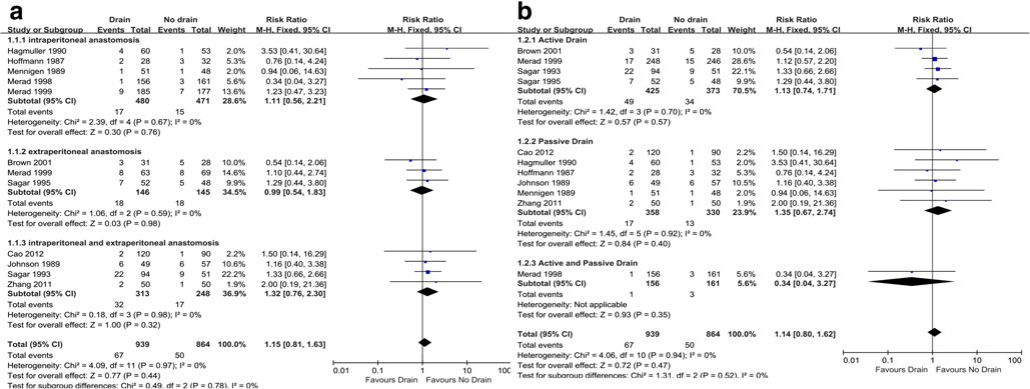
Forest plots of the relative risk (RR) for overall anastomotic leakage, stratified by **a** the site of anastomosis and **b** the type of drainage

All eleven trials reported this outcome. There was no statistical significant difference in the occurrence of overall anastomotic leakage between the drain group and the no drain group (11 trials, *n* = 1803, RR = 1.14, 95 % CI 0.80–1.62, *P* = 0.47) [15–25]. There was no heterogeneity among trials (*Ι*^2^ = 0 %). Through stratifying by sites of anastomosis, we found no significant difference between the subgroup of intraperitoneal anastomosis (5 trials, *n* = 951, RR = 1.11, 95 % CI 0.56–2.21, *P* = 0.76) [17, 18, 20–22] and extraperitoneal anastomosis (3 trials, *n* = 291, RR = 0.99, 95 % CI 0.54–1.83, *P* = 0.98; Fig. 4a) [15, 22, 24]. Stratification by types of drainage showed no benefit of drain for both active drain (4 trials, *n* = 798, RR = 1.13, 95 % CI 0.74–1.71, *P* = 0.57) [15, 22–24] and passive drain (6 trials, *n* = 688, RR = 1.35, 95 % CI 0.67–2.74, *P* = 0.40; Fig. 4b) [16–20, 25]. We also stratified the enrolled participants according to the race into Asian (3 trials, *n* = 369) [15, 16, 25] and European (8 trials, *n* = 1434) [17–24]. The RRs were 0.89 (95 % CI 0.32–2.43, *P* = 0.82) and 1.18 (95 % CI 0.81–1.72, *P* = 0.39), respectively, indicating no significant difference.

#### Clinical anastomotic leakage

**Fig. 5:**
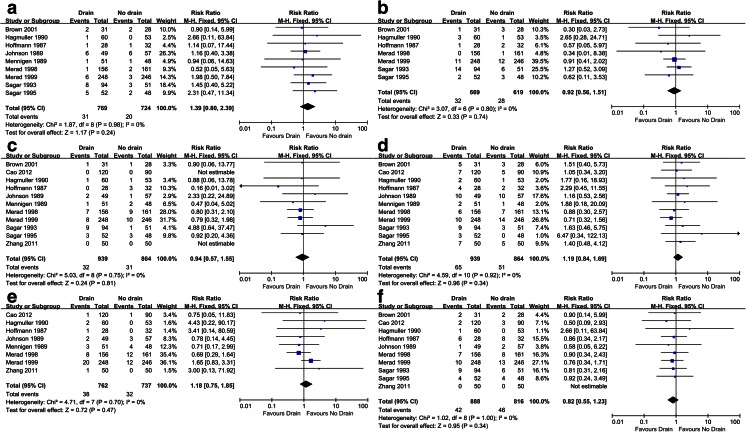
Forest plots of the relative risk (RR) for **a** clinical anastomotic leakage, **b** radiologic anastomotic leakage, **c** mortality, **d** wound infection, **e** re-operation, and **f** respiratory complications

Nine trials reported this outcome [15, 17–24]. There was no statistically significant difference between two groups (9 trials, *n* = 1,493, RR = 1.39, 95 % CI 0.80–2.39, *P* = 0.24). We used a fixed-effect model because no heterogeneity existed (*Ι*^2^ = 0 %; Fig. 5a).

#### Radiologic anastomotic leakage

Seven trials reported this outcome [15, 17, 18, 21–24]. There was no statistically significant difference between the drain group and the no drain group (7 trials, *n* = 1288, RR = 0.92, 95 % CI 0.56–1.51, *P* = 0.74). Heterogeneity was not found (*Ι*^2^ = 0 %; Fig. 5b). A fixed-effect model was used.

### Secondary outcomes

There was no statistical significant difference between the drain group and the no drain group in the following secondary outcome measures:

#### Mortality

Eleven trials, *n* = 1803, RR = 0.94, 95 % CI 0.57–1.55, *P* = 0.81. There was no between-trial heterogeneity (*Ι*^2^ = 0 %; Fig. 5c);

#### Wound infection

Eleven trials, *n* = 1,803, RR = 1.19, 95 % CI 0.84–1.69, *P* = 0.34. There was no between-trial heterogeneity (*Ι*^2^ = 0 %; Fig. 5d);

#### Re-operation

Eight trials, *n* = 1499, RR = 1.18, 95 % CI 0.75–1.85, *P* = 0.47. There was no between-trial heterogeneity (*Ι*^2^ = 0 %; Fig. 5e);

#### Respiratory complications

Ten trials, *n* = 1704, RR = 0.82, 95 % CI 0.55–1.23, *P* = 0.34. There was no between-trial heterogeneity (*Ι*^2^ = 0 %; Fig. 5f).

### Sensitivity analyses

In the sensitivity analysis, a trial not reporting randomized scheme and allocation concealment [16] and a trial using patient’s year of birth to randomize [19] were excluded. The analyses showed that both the primary and the secondary outcomes did not favor the drain group (Supplementary Fig. 1).

### Subgroup analyses

We performed subgroup analyses based on the site of anastomosis (intraperitoneal, 5 RCTs, 951 patients and extraperitoneal, 3 RCTs, 291 patients), the type of drainage (active, 4 RCTs, 798 patients and passive, 6 RCTs, 688 patients), and the race (Asian, 3 RCTs, 369 patients and European, 7 RCTs, 1434 patients). In three subgroup analyses, no statistical significance was found both in the primary and the secondary outcomes between the drain group and the no drain group (Supplementary Fig. 2 and Figs. 3 and 4).

### Assessment of publication bias

**Fig. 6:**
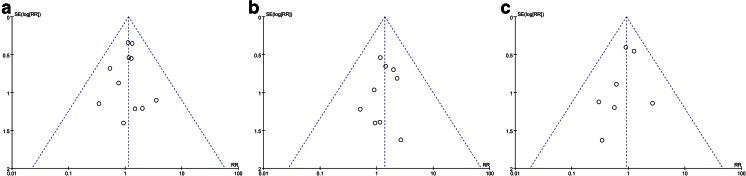
Funnel plots of **a** overall anastomotic leakage, **b** clinical anastomotic leakage, and **c** radiologic anastomotic leakage. *RR* relative risk, *SE* standard error

We performed the funnel plot analysis for the outcomes and observed no obvious asymmetry (Fig. 6). Therefore, we concluded that there is no significant publication bias about these outcomes in the included trials.

## Discussion

This meta-analysis sought to determine whether prophylactic placement of a drain after colorectal anastomosis could reduce the anastomotic leakage rate and other complications. We included 1803 patients of eleven randomized controlled trials in this meta-analysis. Our results showed that using a prophylactic drain did not reduce overall anastomotic leakage rate and using a drain was not associated with reduced risk of clinical and radiologic anastomotic leakage. In addition, we found no significant differences in the occurrence of mortality, wound infection, re-operation, and respiratory complications between the drain group and the no drain group. The sensitivity analyses also indicated no statistical significance in the primary and secondary outcomes between the drain group and the no drain group after excluding two low-quality trials.

For some surgeons, the main purpose of using a drain after colorectal anastomosis is to guide exudation to flow out of abdominal cavity rather than accumulation, in case of anastomotic dehiscence and infection [26]. Anastomotic leakage, hemorrhage, or infection of abdominal cavity are expected to be diagnosed early by prophylactic placement of a drain [26, 27]. Nonetheless, the surgeons who opposed routine use of a prophylactic drain claimed that it could cause infection [6, 28], stimulate the formation of serous fluid [29], and get blocked quickly [30]. A meta-analysis conducted by Urbach et al. showed that only in 1 of 20 clinical leaks did pus or enteric content actually appear in the effluent of the existing drain [31]. It seems that once a leakage appeared, drains cannot guide the leakage substance out of the abdominal cavity completely. Therefore, relying only on a drain to detect anastomotic leakage may give surgeons a false sense of security. In addition, prophylactic drainage showed no benefit in several types of operations such as pancreatic re-section [32], pelvic lymphadenectomy and hysterectomy [33], breast cancer [34], lumbar spine surgery [35], and hip and knee arthroplasty [36]; thus, the results of this meta-analysis can provide a guideline for surgeons about routine prophylactic placement of drains after colorectal anastomosis. Although it shows that there was no statistical heterogeneity in the meta-analysis, potential clinical heterogeneities may exist, such as the different site of anastomosis, drainage materials, and participants. Through stratifying the RCTs based on the site of anastomosis (intraperitoneal and extraperitoneal), the type of drainage (active drain with suction and passive drain without suction), the race (Asian and European), and excluding two low-quality trials, subgroup analyses and sensitivity analyses were performed to address the potential clinical heterogeneities.

The presacral space is non-peritonealized and the fluid absorption and anastomotic healing is slower than peritonealized area [37]. The incidence of leakage is higher when the anastomosis is distal and extraperitoneal as previously reported [38–41]. Thus, some surgeons believed that the drainage of extraperitoneal anastomosis may have potential benefit after rectal excision. Indeed, the Dutch total mesorectal excision (TME) trial provided support for using pelvic drainage in decreasing anastomotic failure rate and the need for re-intervention [42]. In our meta-analysis, intraperitoneal anastomosis studies and extraperitoneal anastomosis studies were analyzed separately, and the results of primary and secondary outcomes between the drain group and the no drain group showed no significant difference. In the Dutch TME trial, decision to place a drain was mainly according to the surgeon’s discretion, not randomization, which may account for a favorable result tendentiously. In addition, a recent prospective study including 978 patients confirmed that pelvic drainage after anterior re-section for primary rectal cancer did not reduce the incidence of anastomotic leakage [43]. Moreover, a randomized study about anterior re-section of the rectum found that fluid collection persisted as late as 7 days postoperatively even when sump drains were used in the pelvis [44]. Pelvic fluid may communicate through the peritoneal cavity, and not all of it can be captured by the drain [45]. Therefore, prophylactic placement of a drain cannot benefit patients with either intraperitoneal anastomosis or extraperitoneal anastomosis.

With regards to the type of drainage, both active drain (drain with suction) and passive drain (drain without suction) subgroups showed no significant difference in anastomotic leakage rates and other complications between the drain group and the no drain group according to our meta-analysis. In addition, suction-irrigation drainage is an independent risk factor of anastomotic leakage after rectal anastomosis while the other types of drainage (silastic drain and silicone flat drain connecting to a vacuum ball) are not associated with this complication [43]. Anastomotic healing could be inhibited by using latex drains and avoiding silastic, polyvinyl chloride (PVC), and Teflon drains [5]. Therefore, in addition to increased cost of hospitalization, doctors who choose to use a prophylactic drainage have to deal with the detrimental effect of drain on the patient’s anastomotic healing if an improper drain is used. Polyurethane caused the least adhesion formation intraperitoneally than Teflon, silicon, and PVC [46]. Currently, a thin-walled tube-like drain called C-seal is developed, which is composed of biodegradable polyurethane and applied with a circular stapler in stapled anastomoses within 15 cm from the anal verge [47]. C-seal does not prevent the formation of dehiscence but precludes extravasation of bowel contents into the peritoneal cavity and gradually degrades to be expelled from the body along with the bowel contents within 10 to 14 days. A multicenter randomized controlled trial is undergoing to evaluate the efficacy of C-seal in reducing anastomotic leakage in stapled colorectal anastomoses [48]. In several cases in which drainage may be required such as the surgery with technical difficulties, uncontrolled bleeding, peritonitis due to perforation, or the surgeon is not confident about the procedure [22, 43]. However, from the meta-analysis and other available evidences, even if surgeons choose to place a prophylactic drain, which type of drainage to use is still an outstanding problem.

In the drain group of all the included trials, drainage time is less than 8 days. Only one of the included trials compared 3- and 7-day placement time of drainage with no drain, and no significant difference was found in the three groups in the incidence of complications and the length of hospital stay [23]. A study reported that when anastomotic leakage is diagnosed clinically, the median postoperative day is 7 days and the median postoperative day is 16 days when diagnosed radiographically [49]. Another systematic review described that the time of detecting anastomotic leakage by contrast radiography ranges from 4 to 14 days after operation [50]. If surgeons expect a drain to provide information about anastomosis, the drain should not be removed in the early postoperative period [49]. A retrospective single arm study conducted by Shingo et al. showed that changes in the drain content which could detect anastomotic leak were observed in 15 (71 %) patients; however, the median duration of the drain placement was 52 days (range 32–169 days). These results presented an acceptable sensitivity of drain in detecting anastomotic leakage compared with the meta-analysis conducted by Urbach et al., who found that only 1 of 20 drains contained pus or enteric content at the time of diagnosis. Nevertheless, the long duration of placement prolonged hospital stay and increased medical costs, and eight patients who developed surgical complications were related to the use of a drain [27]. Therefore, surgeons should balance the benefit and risk of using a drain. If used, the drain must be placed in the appropriate anastomotic location and observed regularly, and the duration of drainage should be appropriate.

Our meta-analysis has several strengths. We did a comprehensive search of the topic and strict quality assessment of the trial methodology according to the recommendations of the Cochrane Collaboration. The participants included came from six countries, and the number was comparatively large. We conducted sensitivity analyses and three subgroup analyses including the site of anastomosis (intraperitoneal and extraperitoneal), the type of drainage (active and passive), and the race (Asian and European), which helped to address the potential issue of clinical heterogeneities as far as possible. All of the included trials were perspective and randomized, which could make the results convincing and robust.

Our meta-analysis has some limitations. The proportion of disease category included was not the same. The drainage regimens and materials between trials may affect the outcomes. The effect of surgeons’ experience and surgical methods on the procedure outcomes is also a concern. Large randomized controlled trials comparing drainage to non-drainage in colorectal anastomosis is necessary in the future.

## Conclusion

Routine use of prophylactic drainage in colorectal anastomosis shows no benefit in reducing postoperative complications.

## Electronic supplementary material

Below is the link to the electronic supplementary material.

Supplementary Fig. 1 Outcomes of sensitivity analyses. Forest plots of the relative risk (RR) for **a** overall anastomotic leakage, **b** clinical anastomotic leakage, **c** radiologic anastomotic leakage, **d** mortality, **e** wound infection, **f** re-operation, and **g** respiratory complications. (GIF 73 kb)

High-resolution image (TIF 1.99 mb)

Supplementary Fig. 2 Outcomes of site of anastomosis subgroup analysis. Forest plots of the relative risk (RR) for **a** overall anastomotic leakage, **b** clinical anastomotic leakage, **c** radiologic anastomotic leakage, **d** mortality, **e** wound infection, **f** re-operation, and **g** respiratory complications. (GIF 144 kb)

High-resolution image (TIF 3.51 mb)

Supplementary Fig. 3 Outcomes of type of drainage subgroup analysis. Forest plots of the relative risk (RR) for **a** overall anastomotic leakage, **b** clinical anastomotic leakage, **c** radiologic anastomotic leakage, **d** mortality, **e** wound infection, **f** re-operation, and **g** respiratory complications. (GIF 141 kb)

High-resolution image (TIF 3.74 mb)

Supplementary Fig. 4 Outcomes of race subgroup analysis. Forest plots of the relative risk (RR) for **a** overall anastomotic leakage, **b** clinical anastomotic leakage, **c** radiologic anastomotic leakage, **d** mortality, **e** wound infection, **f** re-operation, and **g** respiratory complications. (GIF 120 kb)

High-resolution image (TIF 2.39 mb)
