# Supplementary figures and images for: To drain or not to drain in colorectal anastomosis: a meta-analysis
Source: Int J Colorectal Dis. 2016 Jan 30;31:951–60. doi: 10.1007/s00384-016-2509-6 (PMC4834107; doi:10.1007/s00384-016-2509-6)

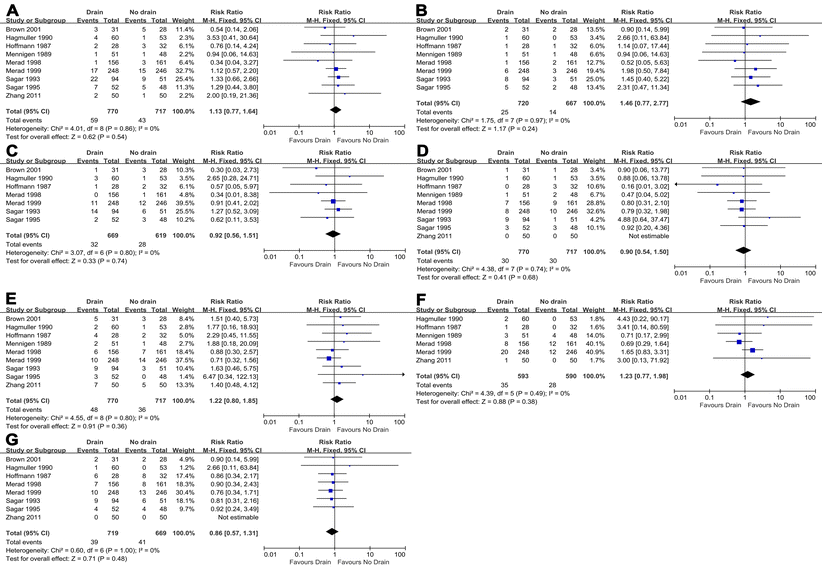

Supplement: Supplementary file 1 — Outcomes of sensitivity analyses. Forest plots of the relative risk (RR) for a overall anastomotic leakage, b clinical anastomotic leakage, c radiologic anastomotic leakage, d mortality, e wound infection, f re-operation, and g respiratory complications. (GIF 73 kb) [file 384_2016_2509_Fig7_ESM.gif]

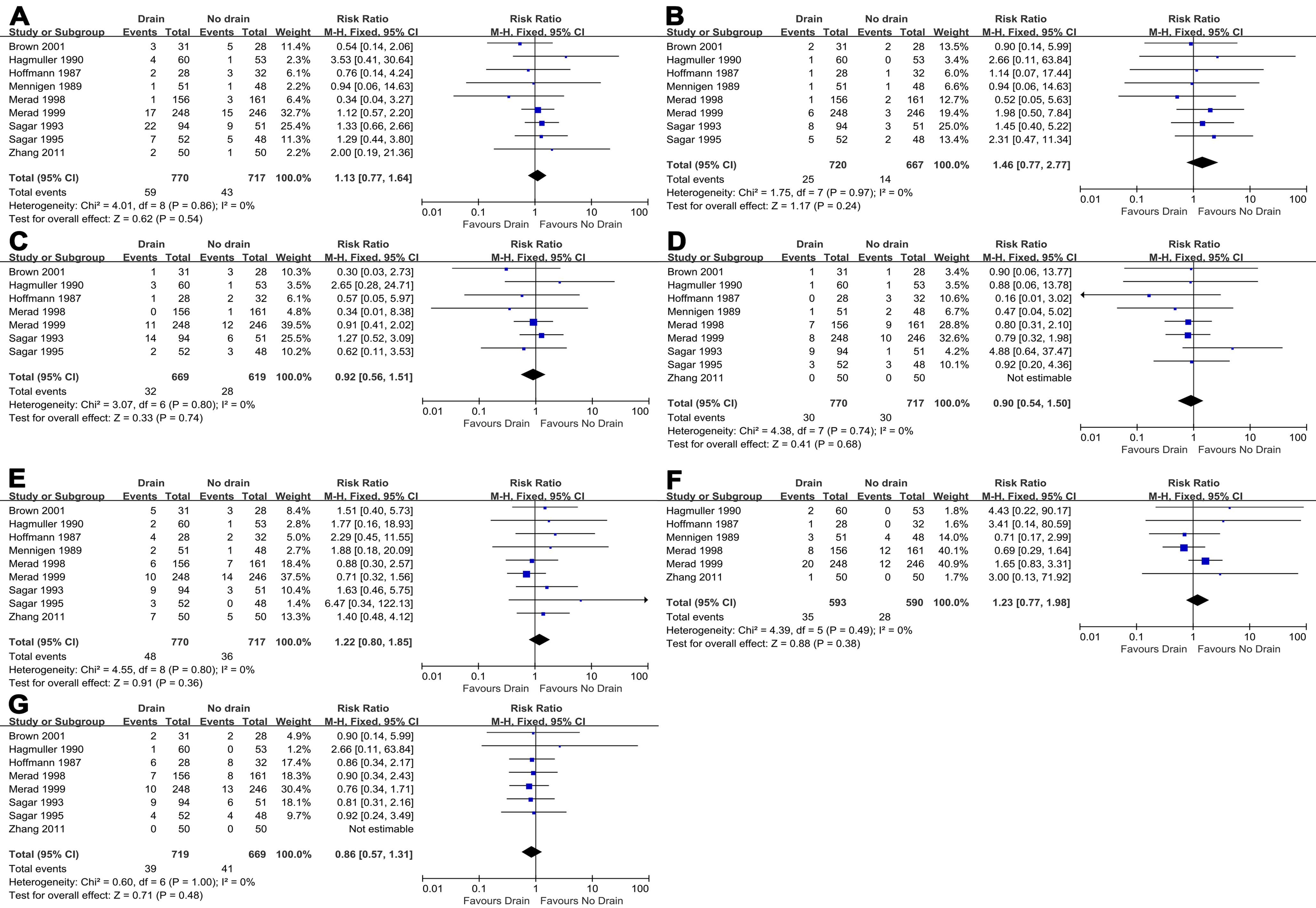

Supplement: Supplementary file 2 — High-resolution image (TIF 1.99 mb) [file 384_2016_2509_MOESM1_ESM.tif]

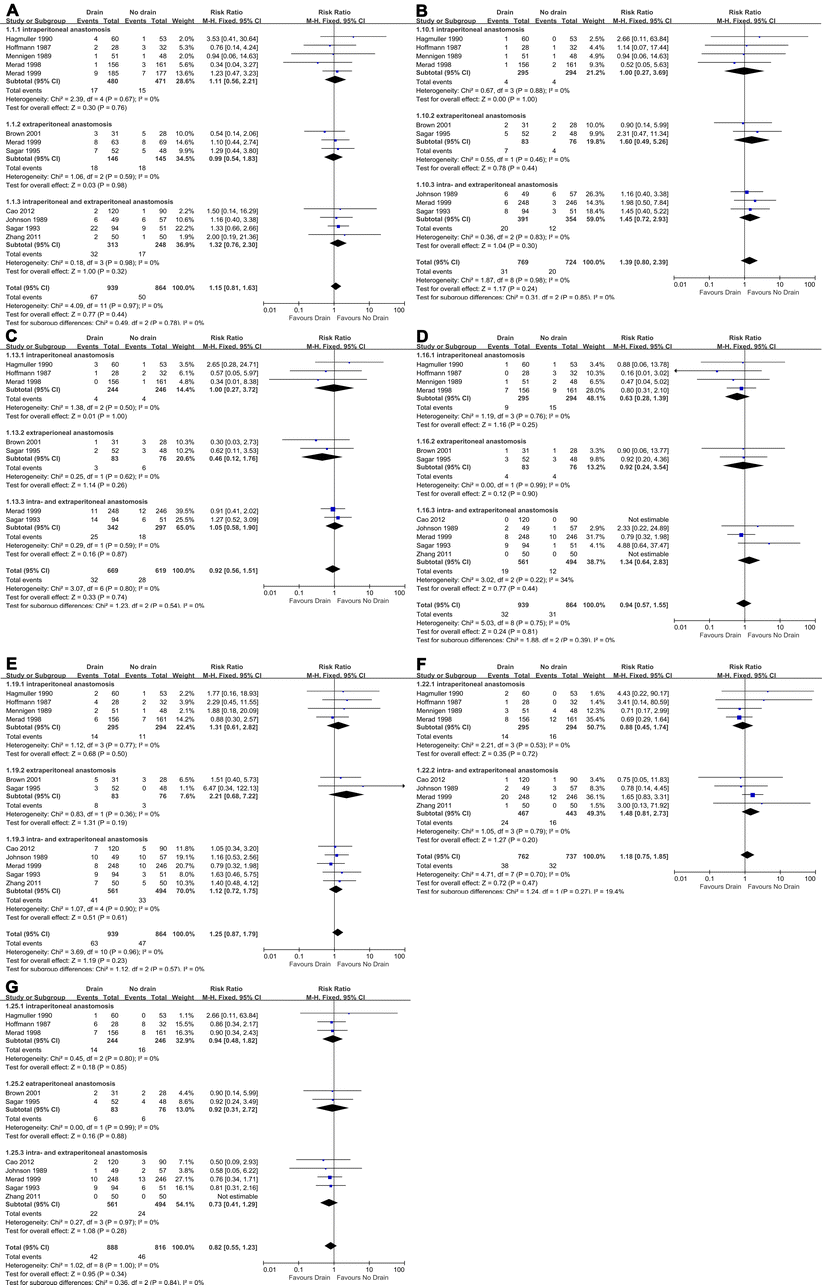

Supplement: Supplementary file 3 — Outcomes of site of anastomosis subgroup analysis. Forest plots of the relative risk (RR) for a overall anastomotic leakage, b clinical anastomotic leakage, c radiologic anastomotic leakage, d mortality, e wound infection, f re-operation, and g respiratory complications. (GIF 144 kb) [file 384_2016_2509_Fig8_ESM.gif]

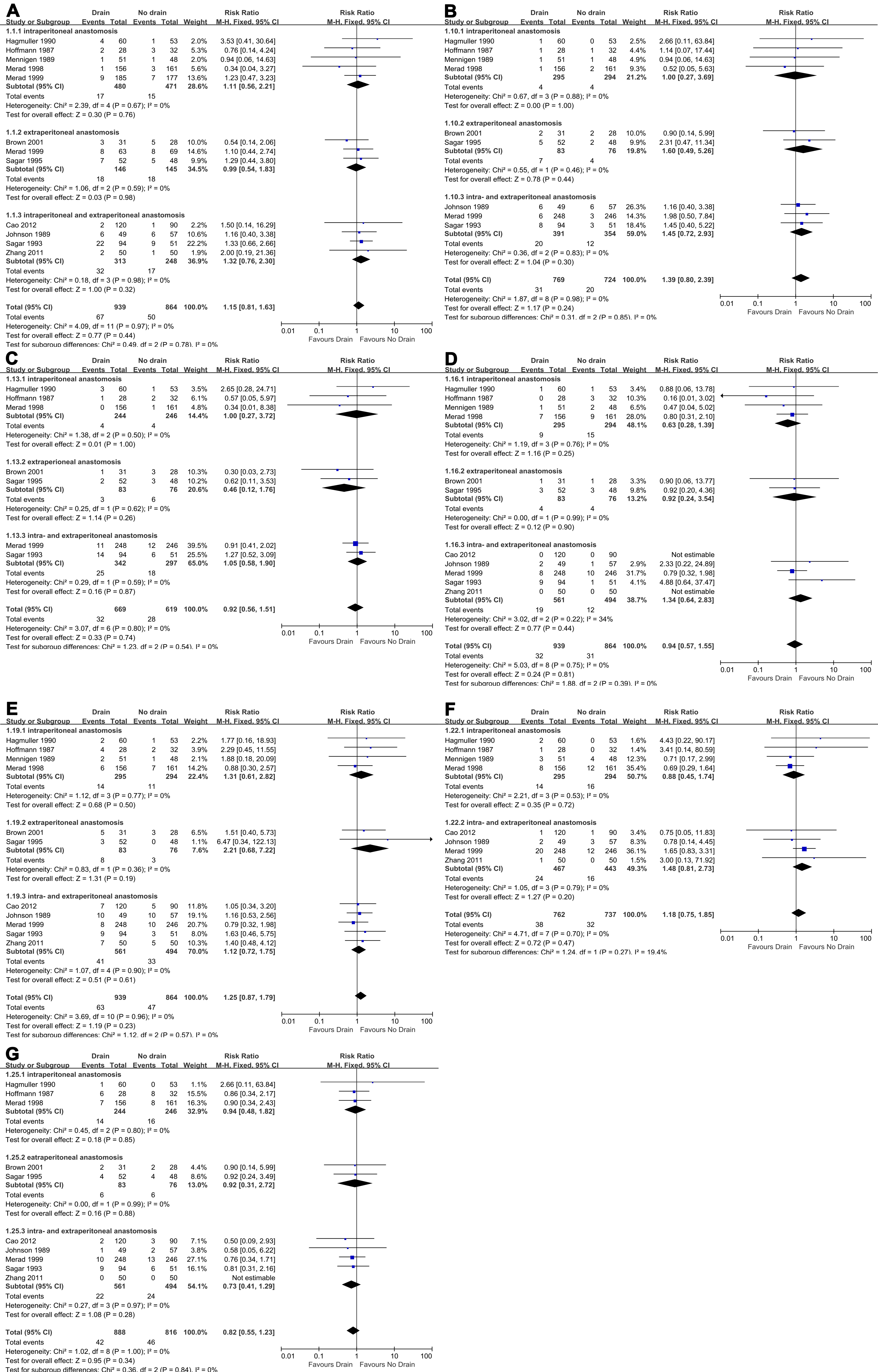

Supplement: Supplementary file 4 — High-resolution image (TIF 3.51 mb) [file 384_2016_2509_MOESM2_ESM.tif]

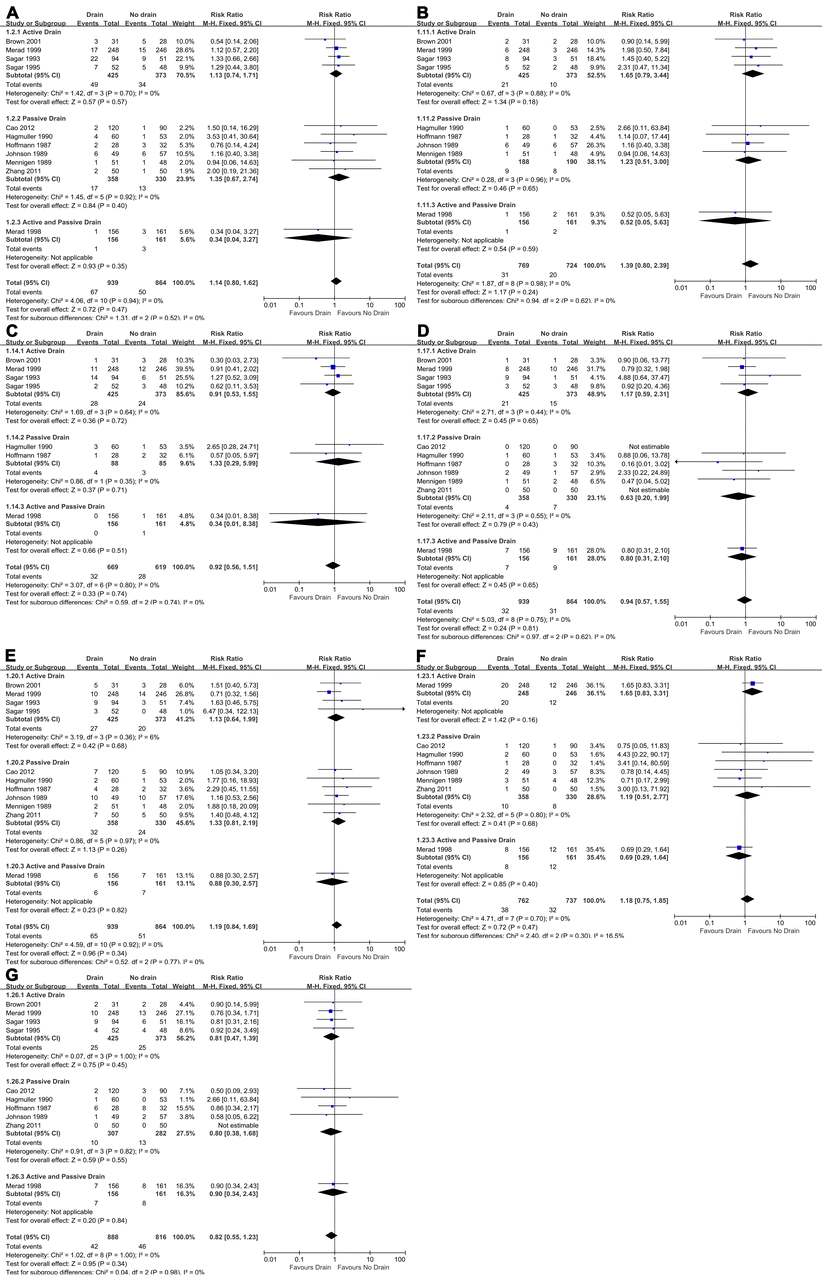

Supplement: Supplementary file 5 — Outcomes of type of drainage subgroup analysis. Forest plots of the relative risk (RR) for a overall anastomotic leakage, b clinical anastomotic leakage, c radiologic anastomotic leakage, d mortality, e wound infection, f re-operation, and g respiratory complications. (GIF 141 kb) [file 384_2016_2509_Fig9_ESM.gif]

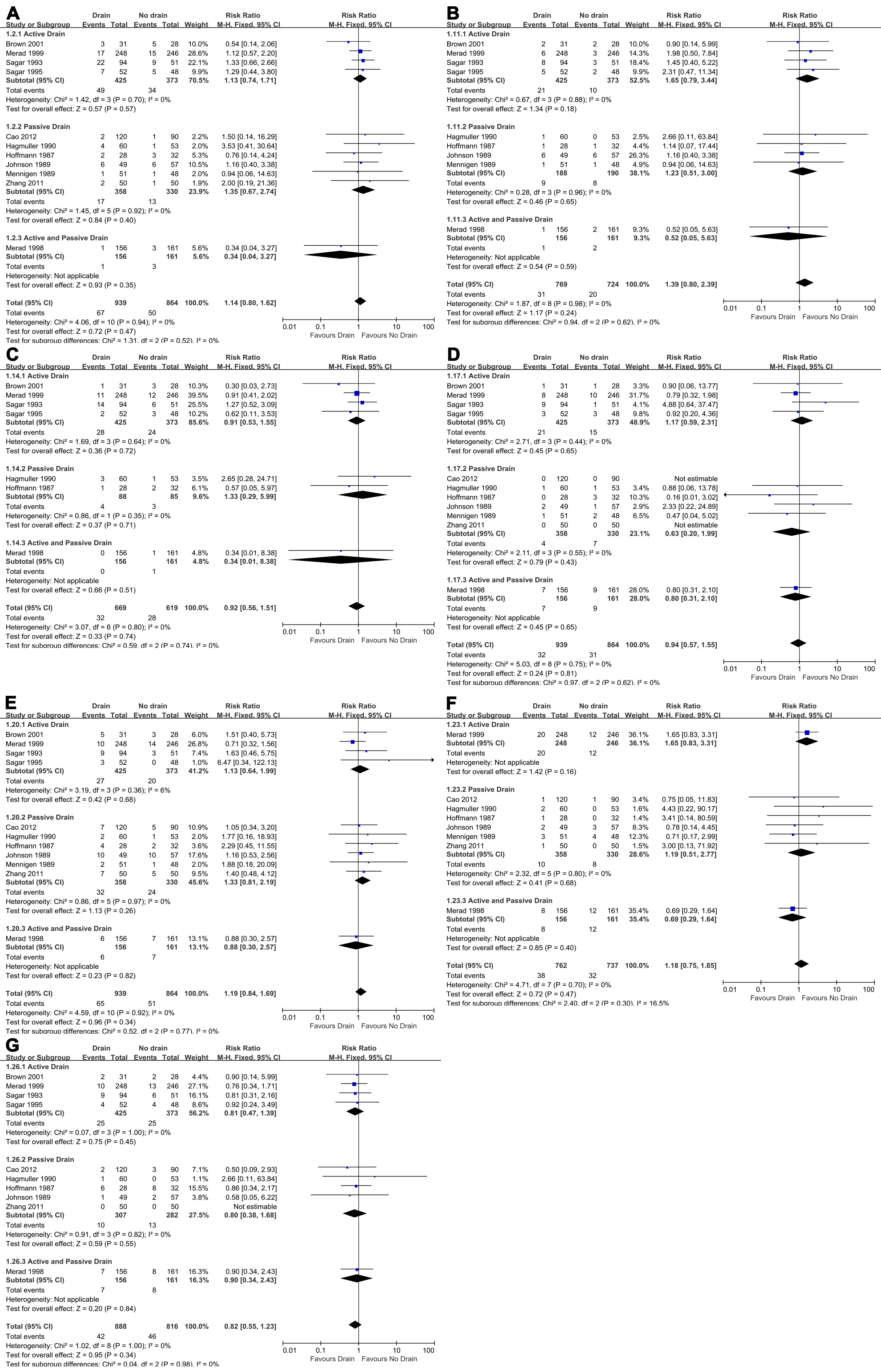

Supplement: Supplementary file 6 — High-resolution image (TIF 3.74 mb) [file 384_2016_2509_MOESM3_ESM.tif]

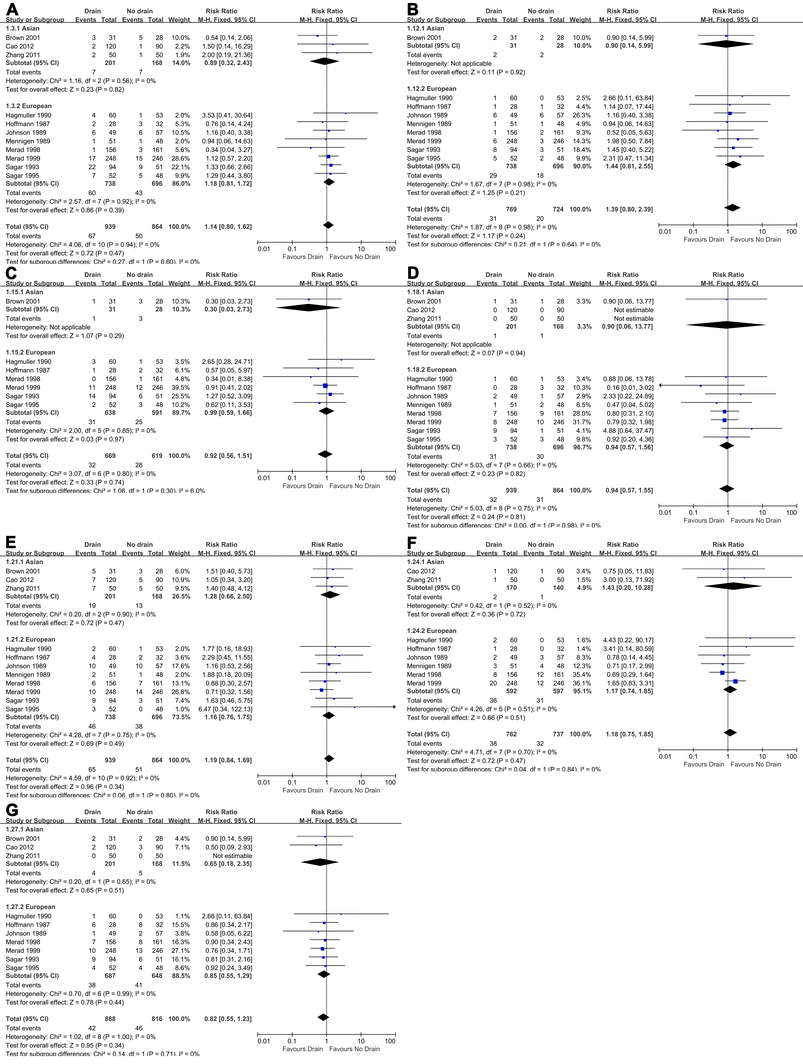

Supplement: Supplementary file 7 — Outcomes of race subgroup analysis. Forest plots of the relative risk (RR) for a overall anastomotic leakage, b clinical anastomotic leakage, c radiologic anastomotic leakage, d mortality, e wound infection, f re-operation, and g respiratory complications. (GIF 120 kb) [file 384_2016_2509_Fig10_ESM.gif]

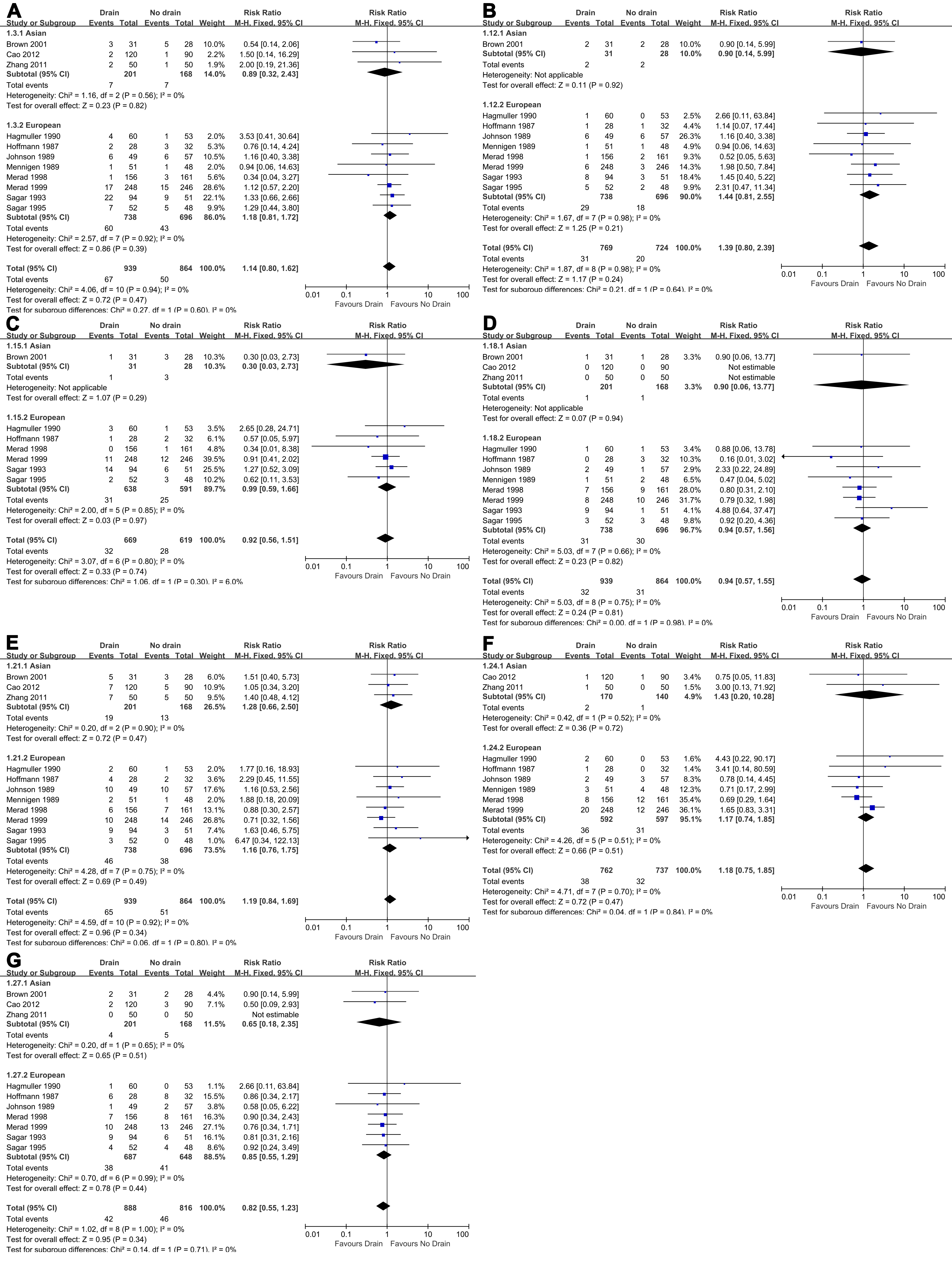

Supplement: Supplementary file 8 — High-resolution image (TIF 2.39 mb) [file 384_2016_2509_MOESM4_ESM.tif]
